# Assessment of the normal and pathological alignment of the elbow in children using the trochleocapitellar index

**DOI:** 10.1186/1471-2474-15-60

**Published:** 2014-02-27

**Authors:** Lauren Gorelick, Dror Robinson, Norman Loberant, Ayala Rozano-Gorelick, Mustafa Yassin, Avraham Garti, Edward Ram

**Affiliations:** 1 Department of Hand Surgery, Rambam Medical Centre, Haifa, Israel; 2 Ruth and Bruce Rappaport Faculty of Medicine, Technion, Haifa, Israel; 3 Department of Orthopedics, Hasharon Hospital, Rabin Medical Centre, Petah Tikwa, Israel; 4 Sackler Faculty of Medicine, Tel Aviv University, Tel Aviv, Israel; 5 Department of Radiology, Western Galilee Hospital, Nahariya, Israel; 6 Leumit Health services, Haifa, Israel; 7 Division of General Surgery, Rabin Medical Centre- Campus Golda, Sackler School of Medicine, Tel Aviv University, Tel Aviv, Israel

**Keywords:** Elbow, Children trauma, Distal Humerus, Physis, Supracondylar fractures

## Abstract

**Background:**

The current research is a retrospective study that involves the description of a new trochleocapitellar index (TCI), on basis of anteroposterior (AP) radiographs of normal and fractured paediatric elbows. This index may be useful in assessing the alignment of the elbow with a supracondylar fracture.

**Methods:**

The index was evaluated to define its normal and pathological range in children between the ages of 1–13 years. A total of 212 elbows in 141 children were radiographically evaluated. 70 children without fracture elbows were evaluated by radiographs taken at the time of trauma. 35 children with unilateral fractures that healed in a normal alignment were compared to 33 patients that had a mal-union and three patients with bilateral elbow fractures. The patients were radiographically assessed at the time of fracture as well as after fracture healing as part of a routine clinical assessment. Treatment included observation, cast or internal fixation as needed.

**Results:**

The current study establishes that the normal range of the TCI was 0.25-0.8. The average TCI is 0.45. The lower range correlates with a valgus alignment of the elbow while the higher range indicates a neutral alignment. The TCI in fractured elbows that have healed in a clinically normal alignment is different than the contra-lateral elbow’s TCI. This might indicate a sub-clinical remaining deformity.

**Conclusions:**

In current practice, paediatric patients with elbow trauma, often undergo bilateral radiographs during emergency room visits. The TCI has high negative and positive predictive values and might be superior to direct angle measurement that is currently in use. The use of the TCI measurement is expected to reduce exposure to irradiation in elbow trauma patients as bilateral comparative films appear to be superfluous when this measurement is used.

## Background

Angulation deformity (cubitus varus and valgus) is the most common complication of displaced supracondylar fractures. Supracondylar fracture tend to leave sequel despite modern treatment methods in up to 46% of cases [1] according to some authors, though this figure might be too high. Most deformity seems to be related to coronal plane angulation [2]. It should also be appreciated that rotation of the distal fragment often worsens varus and valgus angulation [3]. The deformity results from two factors: primary mal-reduction of the fracture, and the limited remodelling in the coronal plane [4]. Prevention of angulation depends on the accurate reduction of the fracture. The gold standard in clinical practice today is assessment of reduction quality using several measurements including Baumann’s angle, the medial epicondylar epiphyseal angle, carrying angle and humerotrochlear angle [2,4]. Baumann angle is formed by the intersection of a line drawn down the humeral axis and a line drawn along the growth plate of the lateral condyle of the elbow [5]. This angle correlates closely with the carrying angle [6]. The mean Baumann’s angle is 72° ±4°. Unfortunately, Baumann’s angle relates only to the coronal plane of a complex rotational deformity of the elbow that involves shifts from the normal anatomy in three planes. The angle is highly dependent on the angulation of the x-ray beam [6]. This is particularly important if there is a variation of the x-ray beam perpendicularity versus the true elbow coronal plane. Thus, in many cases the contralateral elbow has to be radiographed to ensure proper fracture fragments alignment.

Use of a radiographic parameter that relates to two angles might reduce the need for bilateral elbow radiographs. The rationale is that an index based on the relationship between two angles is expected to be less influenced by the radiographic technique and might allow precise evaluation of supracondylar fractures in children.

The object of the current study is evaluation of the possible usefulness of a new radiographic index, in which the relationship between two angles is assessed. This index is termed trochleocapitellar index (TCI) of the elbow. The current data involves children between the ages of one year and thirteen years. In order to assess its potential usefulness, a normal and pathological range was defined based on clinical and radiological correlation. Several groups of patients were assessed. The first group included patients with elbow trauma, in whom radiographs were taken due to suspicion of a possible fracture. This was ruled-out after radiographic assessment. The second group included patients with healed supracondylar fracture with residual deformity (both varus and valgus deformities). The third group consisted of patients with a healed supracondylar fracture without any residual deformity. This retrospective study has been approved by the Rambam hospital institutional ethical review board as well as the head of radiology department and the hospital general manager. The oral consent process by the legal guardian for radiographs was obtained according to hospital standardized operating procedures and approved by the hospital administration and ethical review board.

## Methods

### Patients’ demographics

The age range of the children in our study was from 1 to 13 years old (mean 6.4 years). This is the age range when most supracondylar fractures occur, as the condition is rare both in infancy and after 13-years of age when the distal humeral physis undergoes fusion [7,8].

### Patient’ grouping

This study evaluated 141 children, divided into 4 groups:

• Group 1 included 70 children with bilaterally normal elbows who were radiographed due to suspicion of possible fracture, during routine evaluation in the emergency department after trauma. Evaluation of the injured side was done clinically and radiographically.

• Group 2 consisted of 35 children with unilateral supracondylar fracture which had healed with clinically normal alignment.

• Group 3 comprised 33 patients with unilateral supracondylar fracture with a varus or valgus deformity. Due to clinical need, in this group both elbows were evaluated at the time of fracture reduction as well as after fracture healing.

• Group 4 comprised only 3 patients with bilateral elbow fractures; these had healed in varus (2 children) and valgus (1 children) bilaterally.

In the latter three groups of patients with fracture, radiographs of both elbows were evaluated at the time of fracture reduction and after healing of the fracture. Clinical correlation was available for each child.

In total 212 elbows were evaluated: 138 of them were normal elbows that had not sustained a fracture. 35 fractured elbows which had healed in clinically normal alignment: and 39 fractured elbows that have healed in varus or valgus.

### Follow up period

All patients treated due to supracondylar fractures were followed up at least for one year (range one to five years). 138 AP radiographs of normal extended elbows were selected to determine the normal range of the new TCI. We evaluated 74 fractured elbows after reduction. If there was internal fixation by K-wires, we used AP radiographs with the elbow in extension. In other types of treatment, we utilized AP radiographs with the elbow in flexion and cephalad-caudad x-ray beam angulation between 30 and 45 degrees. All fractures were followed after healing using AP radiographs in extension to confirm that they had healed either with or without angular deformity.

## The trochlear and capitellar angles

**Figure 1:**
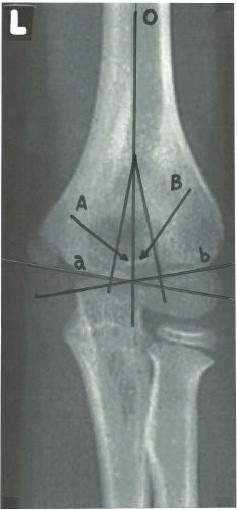
**Schematic drawing of anteroposterior elbow radiograph showing the components of the trochleocapitellar index.** O–humeral axis; A-trochlear angle; B-capitellar angle; a- Trochlear line; b- Capitellar line.

**Figure 2:**
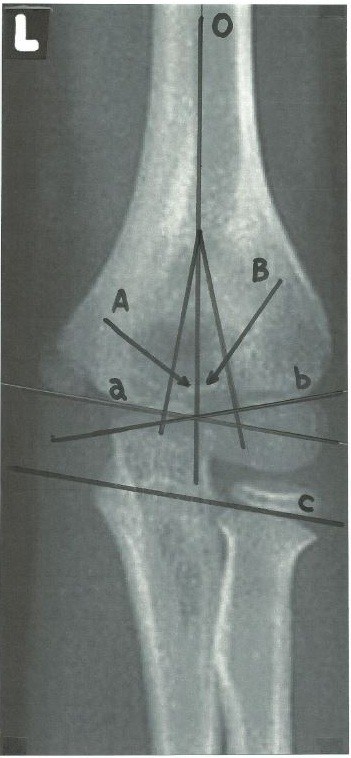
**Schematic drawing of antero-posterior elbow radiograph showing the alternative measurement of the trochlear angle.** This method employs a line drawn through the redial head growth plate and coronoid which is parallel to the trochlear line. O- humeral axis; A- trochlear angle; B- capitellar angle; a- trochlear line; b- capitellar line; c- a line drawn through the radial head growth plate and coronoid which is parallel to the trochlear line.

**Figure 3:**
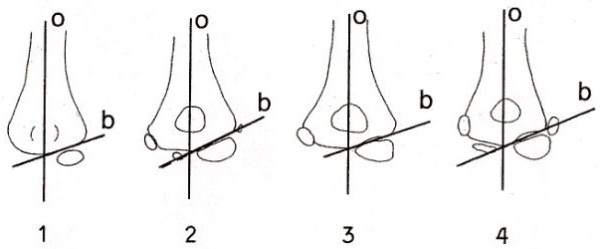
The capitellar line is consistent between the ages of 1 and 13 years of age despite changes in overall appearance of the elbow due to development of secondary and tertiary ossification centres.

On the AP view two important angles were defined: trochlear angle and capitellar angle. The trochlear angle is created by intersection of a line drawn down the humeral axis and a line perpendicular to one drawn along the trochlear surface. The capitellar angle is created by intersection of a line drawn down the humeral axis and one perpendicular to a line drawn along the growth plate of the lateral condyle (Figure 1). For both angles, it is important that the intersection point is proximal to the supracondylar area, preferably is should be located proximal to the olecranon fossa. The intersection point may be identical for the two angles. Sometimes the determination of the trochlear surface line is difficult in children under 3 years of age due to the rounded configuration of the medial humeral metaphysis [9]. In some children there may be some difficulty determining the trochlear line due to the bony prominence of medial aspect of the trochlea; this prominence results from the spiral orientation of the trochlea, and is not part of the plane of joint movement, and is therefore excluded from measurement. In these cases of difficulty in measuring the trochlear line directly, we recommend using a different line that is drawn through the coronoid process and parallel to the radial head growth plate (Figure 2). The rationale for the use of this alternative method of determining the trochlear line is based on two anatomical facts. First, this line must be parallel to the trochlear surface line, since the plane of the trochlea must correspond to the plane of the anterior articular surface of the olecranon and articular surface of the radial head. This is supported by the fact that the angular valgus alignment of the humerus with the forearm (the carrying angle) is always a reflection of the oblique angle between trochlea and humerus, and the oblique angle between ulna and olecranon [10]. Secondly, even though there is lateral angulation of about 12° of the redial head to the radial shaft [11], the proximal radio ulnar joint has a very exacting congruence [10], thus the oblique plane through the trochlea corresponds to the oblique plane through the articular surface of the radial head and the anterior articular surface of the olecranon. Regarding the lateral aspect of the elbow, there is generally no difficulty drawing a line through the growth plate of the lateral condyle, despite the various possible configurations of the distal lateral humeral condyle (Figure 3). Some deviation of this line is not significant since the TCI relies not on direct angle measurement but rather on the relationship between two angles and this relationship is maintained.

## The trochleocapitellar index (TCI)

Definition of the normal TCI, trochlear and capitellar angles was performed on 138 AP radiographs of normal extended elbows.

The TCI of the normal elbow is the ratio between the smaller trochlear and larger capitellar angles of the measured elbow.

The trochlear angle of most normal elbows was about half the capitellar angle in this study.

Our study included 39 elbows in which a supracondylar fracture had healed with a proven varus or valgus deformity. In some of these cases, the trochlear and capitellar lines are displaced from the humeral axis. The deformity may be severe enough to cause one of the lines to be displaced to the humeral axis or even to the opposite side (≤0°).

In these cases, the TCI was defined with the angle arbitrarily defined as 1°. This arbitrary definition was decided upon in order to avoid a negative TCI.

The TCI for 35 fractured elbows that healed in clinically correct alignment was measured as well and compared with the TCI of the contralateral normal elbow.

## Predictive value of the TCI

In this study normal clinical alignment after healing was considered as the ‘gold standard’ endpoint. A “true negative” is the event that the test makes a negative prediction, and the subject has a negative result under the gold standard which is clinical examination, and a “false negative” is the event that the test makes a negative prediction, and the subject has a positive result under the gold standard. A true negative in the context of this study is thus a normal TCI in the setting of normal clinical alignment at healing. False negative is defined as normal TCI in the setting of abnormal clinical alignment. A true positive is thus an abnormal TCI in the setting of abnormal clinical alignment at healing, and a false positive is defined as abnormal TCI in the setting of normal clinical alignment. Similar definitions were previously used in the setting of elbow fracture studies [12].

The predictive value is defined as either a Positive predictive value or a negative one. The Negative Predictive Value is defined as the number of true negatives divided by the number of true negatives plus the number of false negatives [12]. A similar formula allows the calculation of the Positive Predictive Value.

## Inter-observer study

We examined inter-observer reliability by giving a set of 29 radiographs to three independent observers. Two senior orthopaedic surgeons and one senior radiologist measured the above described indices and angles on all x-rays.

## Consent statement

All treatment of children including clinical examination and radiological examination has been according to routine clinical treatment norms at the relevant institute. Oral consent has been obtained from parents of children participating in this study, regarding study participation and analysis of clinical data and radiological data, obtained during routine treatment of these children.

## Results

### Trochleocapitellar index of the normal elbow

The mean normal TCI was 0.45, with range of 0.25 to 0.8. Clinical correlation of the measured TCI as compared with clinical measurement of the carrying angle was performed. A TCI closer to 0.25 indicates slight valgus of the elbow. A TCI closer to 0.8 tends toward a neutral position. A TCI that is greater than 0.8 is exceptional in un-fractured elbows. A TCI between 0.8 and 0.9 is rare, accounting for only 1.5% of cases (2 out of 138). In order to draw causative conclusions rather than merely associations between variables, the authors used statistical tests that aim to evaluate effects.

### Trochleocapitellar index of the normally healed fracture

For the 35 elbows that had been fractured and then healed in acceptable alignment, the TCI was between 0.25 and 0.8. However, the TCI was different than the contralateral normal elbow (p < 0.0001, Z = -4.4783).

### Trochleocapitellar index of cubitus varus elbows

**Figure 4:**
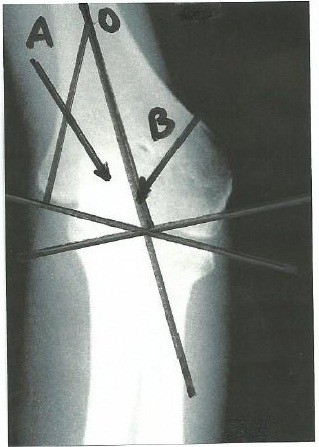
**Follow-up radiographs 5 years after fracture of a cubitus varus elbow.** The TCI is greater than 1.

**Figure 5:**
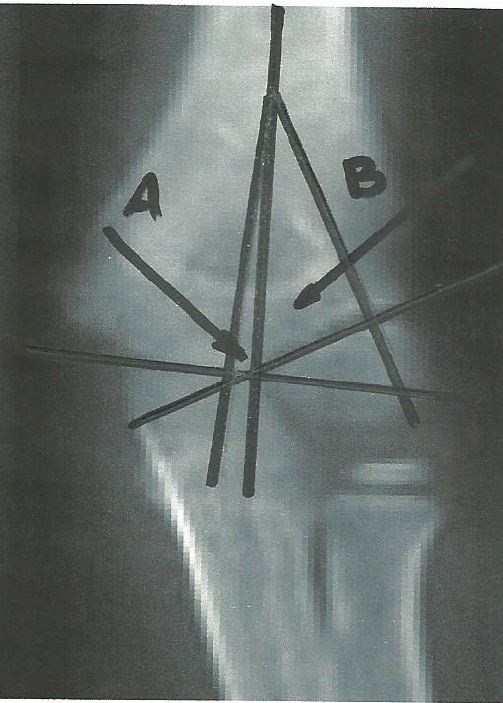
**Follow-up radiographs 2 years after fracture of a cubitus valgus elbow.** In this case the TCI is less than 0.18.

All 27 elbows that were determined to be in cubitus varus had a TCI greater than 1 on postoperative and follow-up radiographs. In severe cases, when the capitellar angle was displaced to the other side of the humeral axis, the TCI was determined as discussed above, by using an arbitrary 1° capitellar angle (Figures 4 and 5). The normal TCI differs significantly from the TCI of cubitus varus elbows (p < 0.00001, Z = -5.1523).

### Predictive value of TCI

The negative predictive value (the likelihood that a normal elbow will have an abnormal TCI) of a normal TCI (normal defined as a ratio between 0.25-0.8) is 98.5% in un-fractured elbows. The positive predictive value of an abnormal TCI for predicting an abnormal clinical alignment is 99%.

### Inter-observer error

In 29 selected radiographs assesse in the inter-observer study, the statistical power was 0.9928. The mean deviation of the index was 0.17 with SD 0.02.

## Discussion

The final assessment of the reduction of a supracondylar fracture in children shows the importance of preventing any angular deformity of the elbow at the time of fracture reduction. The remodelling capacity is limited at the elbow and incapacitating limitation of joint range of motion often occurs due to sub-optimal fracture fragments reduction. Several angels could be measured on AP radiographs in order to allow determination of the degree to which the normal alignment of the elbow has been restored [2-4,6,10]. Unfortunately these measurements are associated with some technical difficulties that lead to difficulty in fracture alignment assessment [13]. Due to these difficulties, it is often clinically mandated to compare the injured elbow with the contralateral elbow [14]. Exposing the contra-lateral elbow to radiation not only increases the radiation exposure of the children but also the cost of treatment. It appears that the TCI might offer a method that does not require contra-lateral elbow exposure to radiation.

Furthermore, the elbow is a highly congruent joint. Thus, small measurement errors might have a clinically important significance. The TCI measurement advantage lies in the measurement of the angulation between both the trochlear and capitellum to the humeral axis. The relationship appears relatively constant in the normal elbow. In the normal elbow the trochlear angle is approximately half the capitellar angle. On the basis of these data, the elbow TCI is determined. In 138 AP radiographs of normal elbows, the mean normal index was 0.45, with range of 0.25-0.8. While this range might appear relatively large, it appears to clearly distinguish between clinically deformed elbows after fracture and the normal elbows.

The TCI appears to have a strong predictive power for discerning abnormal elbow position after reduction as it was found to be abnormal in all of the 27 cubitus varus and all of the 12 cubitus valgus elbows.

Another advantage of the TCI measurement as compared with other published measurements is that lateral or medial displacement, as well as rotation of the distal fragment affects the relationship between the two angles.

The authors suggest that in some severe cases, one angle might become zero or negative (i.e. extreme deviation to the “wrong” side of the axis). In those infrequent cases, we recommend that an angle less than or equal to zero be arbitrarily set at 1 degree to prevent the necessity of dealing with negative TCI. In this study the TCI of all varus-misaligned elbows was greater than 1. All valgus-misaligned elbows were less than 0.2.

The 35 fractured elbows that were healed with normal alignment showed normal TCI. However, the index was never the same as that of the contralateral side. We suggest that when the TCI is within the normal interval (0.25-0.8), elbow alignment after reduction is normal. The difference between the normal side and the functionally unimpaired healed elbow might represent a clinically undetectable abnormality of the elbow.

The above reported results appear to indicate that the normal range for the TCI is constant in children between the ages of 1 to 13 years and does not vary in this age range. The TCI shows low inter-observer variability. The advantage might be that it is less dependent on direct measurement of angels than other predictors of elbow alignment. It appears that radiographic evaluation of the contralateral elbow is seldom necessary provided the TCI value is within the suggested normal range. In addition, the TCI measurement appears to be less sensitive to sagittal plane variation of the x-ray beam alignment relative to the elbow. This feature of the new index is helpful in cases were full elbow extension is not possible.

## Conclusions

The TCI is a new index representing the relationship between the smaller trochlear and lager capitellar angles of the measured elbow. The mean normal index was 0.45, with range of 0.25-0.8, in normal elbows. The TCI is less dependent on direct measurement of angels than other predictors of elbow alignment. The authors recommend that provided the TCI is within the normal range there is no need for comparison with the contralateral elbow.

It appears that the TCI might serve as another and possibly more definitive predictor of physis alignment in supracondylar fractures of the elbow and assist accurate reduction achievement.

## Competing interests

The authors declare that they have no competing interests.

## Authors’ contributions

LG conceived of the study, and participated in its design and coordination and helped to draft the manuscript, DR analyzed the data and edited the manuscript, NL and ARG carried out the study. MY and AG have edited the manuscript, ER participated in the study and manuscript writing and performed the statistical analysis. All authors read and approved the final manuscript.

## Pre-publication history

The pre-publication history for this paper can be accessed here:

http://www.biomedcentral.com/1471-2474/15/60/prepub
